# Validation of the Predictive Model of the European Society of Cardiology for Early Mortality in Acute Pulmonary Embolism

**DOI:** 10.1055/s-0038-1669427

**Published:** 2018-09-06

**Authors:** Massimo Cugno, Federica Depetri, Laura Gnocchi, Fernando Porro, Paolo Bucciarelli

**Affiliations:** 1 Division of Internal Medicine, Department of Pathophysiology and Transplantation, Università degli Studi di Milano, Milan, Italy; 2 Angelo Bianchi Bonomi Hemophilia and Thrombosis Center, Fondazione IRCCS Ca' Granda Ospedale Maggiore Policlinico, Milan, Italy; 3 Emergency Medicine Unit, Fondazione IRCCS Ca' Granda Ospedale Maggiore Policlinico, Milan, Italy

**Keywords:** pulmonary embolism, mortality predictive model, D-dimer

## Abstract

**Background** Acute pulmonary embolism (PE) is burdened by high mortality, especially within 30 days from the diagnosis. The development and the validation of predictive models for the risk of early mortality allow to differentiate patients who can undergo home treatment from those who need admission into intensive care units.

**Methods** To validate the prognostic model for early mortality after PE diagnosis proposed by the European Society of Cardiology (ESC) in 2014, we analyzed data of a cohort of 272 consecutive patients with acute PE, observed in our hospital during a 10-year period. Moreover, we evaluated the additional contribution of D-dimer, measured at PE diagnosis, in improving the prognostic ability of the model. All cases of PE were objectively diagnosed by angiography chest CT scan or perfusion lung scan.

**Results** The overall mortality rate within 30 days from PE diagnosis was 10% (95% confidence interval [CI]: 6.4–13.5%). According to the ESC prognostic model, the risk of death increased 3.23 times in the intermediate-low-risk category, 5.55 times in the intermediate-high-risk category, and 23.78 times in the high-risk category, as compared with the low-risk category. The receiver operating characteristic analysis showed a good discriminatory power of the model (area under the curve [AUC] = 0.77 [95% CI: 0.67–0.87]), which further increased when D-dimer was added (AUC = 0.85 [95% CI: 0.73–0.96]).

**Conclusion** This study represents a good validation of the ESC predictive model whose performance can be further improved by adding D-dimer plasma levels measured at PE diagnosis.

## Introduction

Pulmonary embolism (PE) is a common cardiovascular disease with high mortality. Among the cardiovascular diseases, it represents the third cause of death after acute myocardial infarction and stroke. 1 2 3 4 Despite the ongoing progress in diagnosis, treatment, and prevention over the last two decades, the number of deaths still remains high, 5 6 representing 9 to 11% of cases of PE in the first 30 days after the acute event. 7

The development of predictive models to define the risk of early mortality and the validation of these models are the goals to be achieved to optimize the management of patients with PE.

In 2014, the European Society of Cardiology (ESC) proposed a prognostic model for early mortality (i.e., within 30 days) after PE diagnosis based on integrated clinical, laboratory, and instrumental parameters, 7 which identifies four categories of early mortality risk: high, intermediate-high, intermediate-low, and low risk. 8 9 The definition of risk category is based on hemodynamic instability, class of Pulmonary Embolism Severity Index (PESI), evidence of right ventricle dysfunction (documented by echocardiography or computed tomography [CT] angiography), and elevated cardiac biomarker levels, that is, troponins or pro-brain natriuretic peptide (proBNP). This model allows to differentiate patients who can undergo home treatment (low-risk category), reducing hospital costs, from those who need admission into intensive care units from the early stages of the acute event (high-risk category). 10 11 However, according to a recent study in which the ability of the 2014 ESC model to predict 30-day mortality was assessed, the risk stratification in patients at intermediate risk requires further improvement. 8

D-dimer is a product of fibrin degradation and increases in the acute phase of venous thrombosis. The measurement of plasma D-dimer has a clear diagnostic role in symptomatic outpatients with suspected PE because of its high negative predictive value. 12 Moreover, when anticoagulation is stopped after the conventional treatment of a first episode of venous thromboembolism, high D-dimer levels have been shown to be a risk factor for a thrombotic recurrence. 13 Finally, the role of D-dimer as a predictive prognostic biomarker for mortality after PE diagnosis has been well demonstrated. 14 15 16

The aim of this cohort study was to validate the predictive model for early mortality after acute PE diagnosis proposed by the 2014 ESC guidelines in a cohort of patients with acute PE recruited in the emergency department of our hospital in a 10-year period. A secondary objective of the study was to assess the additional contribution of the D-dimer, measured at PE diagnosis, in improving the prognostic ability of the model.

## Patients and Methods

### Study Population and Diagnostic Tests

We included in the cohort 272 patients with acute PE (117 men and 155 women, median age: 65 years [min–max: 24–92 years]) who attended the emergency department of the Ospedale Maggiore Policlinico, University of Milan, in the period between January 2005 and May 2015. All cases of PE were objectively diagnosed by angiography chest CT scan or perfusion lung scan. We excluded patients younger than 18 years, patients who refused to give their consent to the collection of their data, and patients lacking of information on 30-day follow-up. For every patient included in the study, a thorough medical history was available as well as a careful physical examination and diagnostic tests.

Concerning medical history, the risk factors for venous thromboembolism were considered, in particular previous episodes of venous thromboembolism, thrombophilia, recent surgery, plaster casts, prolonged immobilization, estrogen therapy, pregnancy, postpartum period, malignancy, obesity, and chronic heart failure. Symptoms at presentation were accurately analyzed, including dyspnea, syncope, shock, hemoptysis, altered mental status, phlebitic pain, and pleuritic or substernal chest pain. We also analyzed vital signs (systolic and diastolic blood pressure, heart rate, respiratory rate, oxygen saturation on room air, and body temperature) and physical signs (cyanosis, signs of deep vein thrombosis (DVT) in a limb, and jugular turgor). Additional diagnostic tests were arterial blood gas analysis, chest X-ray, electrocardiography, echocardiography, and laboratory parameters (D-dimer, troponin I, proBNP, and lactate dehydrogenase). In particular, an immunoturbidimetric method (D-Dimer HS kit; Instrumentation Laboratory, Bedford, Massachusetts, United States) was used for the quantitative determination of D-dimer in human-citrated plasma (blood collected into vacuum tubes containing 3.2% sodium citrate), following manufacturer's instructions. Lower limb ultrasonography was also performed to search for DVT as a cause of PE.

### Stratification of Early Mortality Risk

The clinical records of the patients were retrospectively analyzed according to the 2014 ESC guidelines. The diagnostic algorithm for risk stratification of early mortality after acute PE diagnosis, proposed by the ESC in 2014, is reported in Table 1 .

**Table 1:** Stratification algorithm for early mortality in patients with acute pulmonary embolism

Early mortality (at 30 d) risk classes		Risk parameters and scores			
		Shock or hypotension	PESI class III–V or sPESI ≥ I	Signs of RV dysfunction on an imaging test	Cardiac laboratory biomarkers
High		+	+	+	+
Intermediate	Intermediate-high	−	+	Both positive	
	Intermediate-low	−	+	Either one (or none) positive	
Low		−	−	Assessment optional; if assessed both negative	

Abbreviations: sPESI, simplified Pulmonary Embolism Severity Index; RV, right ventricle.

For the definition of risk stratification, clinical parameters, PESI score, serum cardiac damage indices, and echocardiography were taken into consideration. PESI score was calculated for each patient, considering 11 clinical parameters detectable at the first visit to the emergency department: age, sex, presence of cancer, heart failure, chronic lung disease, heart rate above 110 beats per minute, systolic blood pressure less than 100 mm Hg, respiratory rate greater than 30 breaths per minute, altered mental status, body temperature less than 36°C, and arterial oxygen saturation below 90%. The model assigns points for each applicable characteristic, and calculates a total score by summing these points and adding the patient's age in years. Serological markers, such as cardiac troponin I and proBNP, were measured at patient arrival and 6 and 12 hours later. Bedside echocardiography was performed to find out right ventricular overload signs and to estimate pulmonary pressure.

All these parameters allowed the definition of risk stratification, dividing patients into four classes:

1. *High-risk class* : patients with shock or hemodynamic instability.
2. *Intermediate-high-risk class* : patients without shock or hemodynamic instability and with PESI greater than II, echocardiographic or tomographic signs of right ventricular overload in addition to altered serum markers of cardiac damage and cardiac dysfunction.
3. *Intermediate* - *low-risk class* : patients without shock or hemodynamic instability and with PESI greater than II and either alteration of cardiac serum markers (troponin I and/or proBNP) or echocardiographic or tomographic signs of right ventricular overload.
4. *Low-risk class* : patients with PESI scores I and II.

Anticoagulant therapy was set up in the whole cohort by administration of unfractionated heparin or low-molecular-weight heparin. Hemodynamically unstable patients were treated with thrombolysis.

From the time of PE diagnosis, patients were followed up for a period of 30 days. The study outcome was death within 30 days, which was confirmed through medical charts or (for patients with early hospital discharge) via a telephone call with their family members or through municipality charts.

The study was conducted following the ethical principles of the Declaration of Helsinki, regulatory requirements, and the code of Good Clinical Practice. The patients or their relatives gave their written informed consent to the collection of their data for scientific purposes.

### Statistical Analysis

All statistical analyses were performed using the statistical packages SPSS, release 24.0 (IBM SPSS Inc., Chicago, Illinois, United States), and R, release 3.0.0 (R Foundation for Statistical Computing, Vienna, Austria). Continuous variables are presented as mean (standard deviation) or median (interquartile range [IQR]), and categorical variables as count and percentage. The nonparametric Kruskal–Wallis test was used for comparison of continuous variables between more than two groups. The prognostic algorithm of early mortality after PE diagnosis, according to the 2014 ESC guidelines, has been validated by means of a logistic regression model, with 30-day mortality (0 = no, 1 = yes) as outcome and the four ESC risk categories (low, intermediate-low, intermediate-high, and high), age (continuous), and sex (0 = female, 1 = male) as predictors. Age- and sex-adjusted odds ratios with 95% confidence intervals (CI) were calculated as estimates of the relative risk of early mortality for all the four ESC risk categories, taking the lowest one as reference. A receiver operating characteristic (ROC) curve was constructed and the area under the curve (AUC) was calculated together with its 95% CI to determine the discriminating power of the analyzed model. To evaluate the additional contribution of D-dimer in the prediction of early mortality after PE diagnosis, a second logistic model was built, which contained D-dimer (categorized at the 75th percentile of its distribution in the whole cohort of patients with acute PE) besides ESC risk categories, age, and sex. The AUC of the ROC curve of this second model was calculated together with its 95% CI. A bootstrap analysis (200 replicates) was performed to correct for optimism. Calibration of the predicted probabilities was assessed by means of graphical methods, plotting them against observed probabilities.

## Results

Of the 272 patients with objectively confirmed acute PE evaluated at the emergency department of our hospital between 2005 and 2015, twenty-seven died within 30 days, for a death rate of 10.0% (95% CI: 6.4–13.5%). In particular, 21 patients died because of PE within 3 days from the acute event and the other 6 patients died within 30 days for heart failure (3), major bleeding (1), embolic recurrence (1), and pneumonia (1). All study subjects were white Caucasians. Baseline characteristics of the whole cohort, divided according to survival status, are shown in Table 2 . Shock, tachypnea, hemoptysis, and DVT signs were more frequent in nonsurvivors (33.3, 63.0, 7.4, and 40.7%, respectively) than in survivors (6.9, 14.3, 2.0, and 26.5%, respectively). Also neoplasia, heart failure, and immobilization were more frequent in nonsurvivors (44.4, 7.4, and 37.0%, respectively) than in survivors (20.4, 3.3, and 19.2%, respectively). Nonsurvivors had also lower levels of systolic blood pressure, pH, PaO _2_ , and oxygen saturation, as well as higher heart rate and respiratory rate, than survivors.

**Table 2:** Baseline characteristics of the 272 patients with acute pulmonary embolism divided according to the survival status at 30 days from the acute event

Features	Survivors *n* = 245	Nonsurvivors *n* = 27
Sex (M/F)	102/143	15/12
Age	69.2 (15.5)	73.0 (13.5)
Neoplasia	50 (20.4)	12 (44.4)
Heart failure	8 (3.3)	2 (7.4)
Surgery (< 4 wk)	21 (8.6)	2 (7.4)
Fractures	20 (8.2)	4 (14.8)
Immobilization	47 (19.2)	10 (37.0)
Dyspnea	189 (77.1)	21 (77.8)
Tachypnea (respiratory rate ≥30)	35 (14.3)	17 (63.0)
Chest pain	78 (32.0)	8 (30.0)
Syncope	76 (31.0)	9 (33.3)
Hemoptysis	5 (2.0)	2 (7.4)
DVT signs	65 (26.5)	11 (40.7)
Shock	17 (6.9)	9 (33.3)
Systolic blood pressure (mm Hg)	129.4 (26.1)	120.2 (33)
Diastolic blood pressure (mm Hg)	75.2 (15.5)	71.6 (17.9)
Heart rate (bpm)	95.2 (18.9)	101.7 (19.7)
Respiratory rate (bpm)	25.4 (6.6)	35 (8.8)
pH	7.5 (0.1)	7.3 (0.3)
PaO _2_ (mm Hg)	64.6 (17.7)	52 (9.4)
PaCO _2_ (mm Hg)	32.4 (7.1)	34 (15.6)
Sat O _2_ (%)	92 (5.6)	82 (12.2)

Abbreviation: DVT, deep vein thrombosis.

Note: Continuous variables are presented as mean (standard deviation) and categorical variables as count (%).

**Fig. 1:**
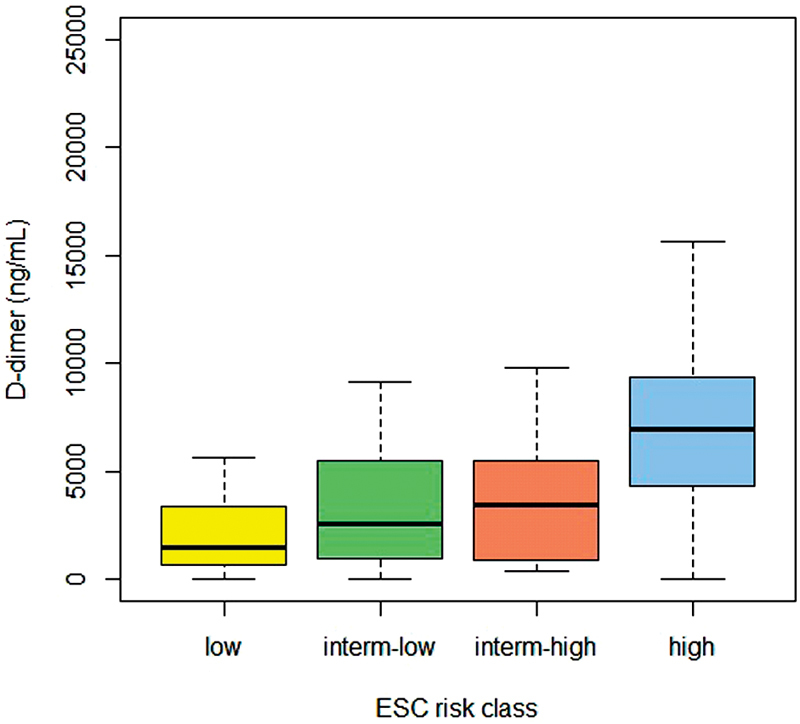
Plasma levels of D-dimer in the four ESC risk classes (low, intermediate-low, intermediate-high, and high). Data are reported as median values, interquartile ranges (boxes), and 2.5th and 97.5th percentiles (whiskers).

PE patients were evaluated according to the algorithm proposed by the 2014 ESC guidelines for definition of a risk class. Thirty-eight patients, all in the group of survivors, were not considered in the analysis of the predictive model because they lacked one or more parameters for the definition of the risk class. The remaining 234 patients (86% of the whole cohort) have been divided into the four ESC classes of risk ( Table 3 ). The mortality was 2.5% (2/81) in the low-risk class, 9.5% (7/74) in the intermediate-low-risk class, 14.8% (8/54) in the intermediate-high-risk class, and 40% (10/25) in the high-risk class. The difference in mortality between intermediate-low- and intermediate-high-risk class was 5.3% (95% CI: −6.3% to 16.9%). The risk of death within 30 days increased progressively with the risk class, being approximately 24-fold higher in the high-risk than in the low-risk class (adjusted odds ratio: 23.78 [95% CI: 4.62–122.0]; Table 4 ). Plasma levels of D-dimer in the four ESC risk classes are shown in Fig. 1 . Median (IQR) levels were 1447 ng/mL (633–3367) in the low-risk class, 2,520 ng/mL (900–5,800) in the low–intermediate-risk class, 3,400 ng/mL (886–5,470) in the high-intermediate-risk class, and 6,972 ng/mL (3,575–9,480) in the high-risk class (Kruskal–Wallis test: *p* = 0.001).

**Table 3:** Risk class of early death (within 30 d after acute PE) calculated at PE diagnosis

	Risk class of death				
	Low	Intermediate-low	Intermediate-high	High	Total
Survivors	79 (38%)	67 (32%)	46 (22%)	15 (7.2%)	207
Nonsurvivors	2 (7.4%)	7 (26%)	8 (30%)	10 (37%)	27

Abbreviation: PE, pulmonary embolism.

Notes: Data obtained from the 234 patients with acute PE (all the 27 nonsurvivors and 207 of the survivors) for whom all parameters for a correct risk classification were available. In each row, percentages are calculated on the total numbers of survivors and nonsurvivors.

**Table 4:** Risk of 30-d mortality after acute PE according to the 2014 ESC risk class

Risk class	Patients, *N* (%)	Adjusted odds ratio (95% CI) a
Low	81 (35)	1 (reference)
Intermediate-low	74 (32)	3.23 (0.60–17.24)
Intermediate-high	54 (23)	5.55 (1.05–29.23)
High	25 (11)	23.78 (4.62–122.0)

Abbreviations: ESC, European Society of Cardiology; PE, pulmonary embolism.

Notes: Data obtained from the 234 patients with acute PE (all the 27 nonsurvivors and 207 of the survivors) for whom all parameters for a correct risk classification were available.

a Odds ratios adjusted for age and sex.

**Fig. 2:**
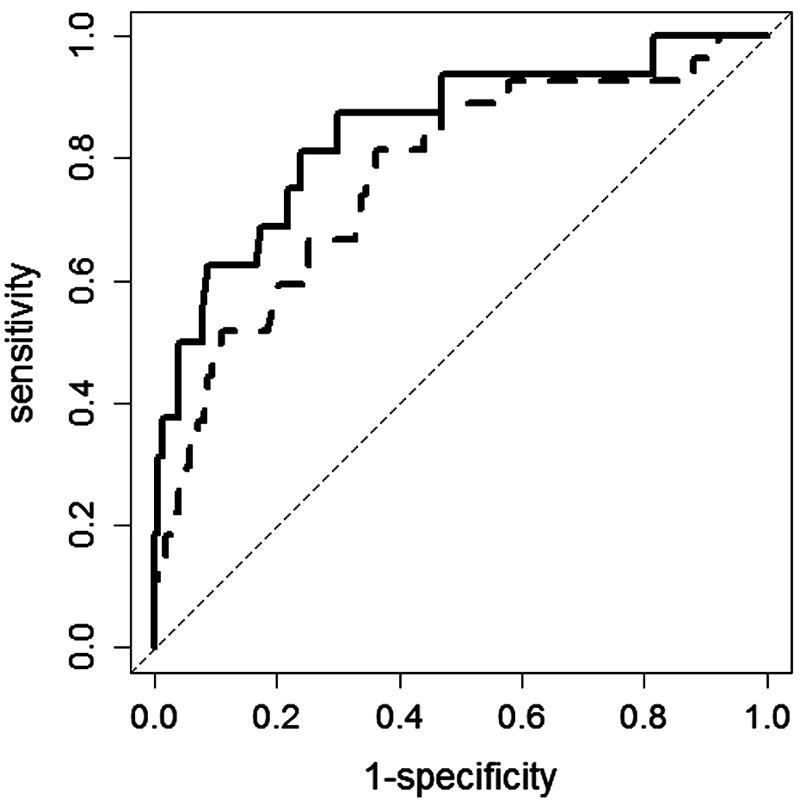
ROC curves of the two predictive models without (dashed line) and with (continuous line) D-dimer, categorized at the 75th percentile of its distribution in the cohort of PE patients (i.e., 5,000 ng/mL). The two models also contained the four ESC risk classes, age, and sex.

**Fig. 3:**
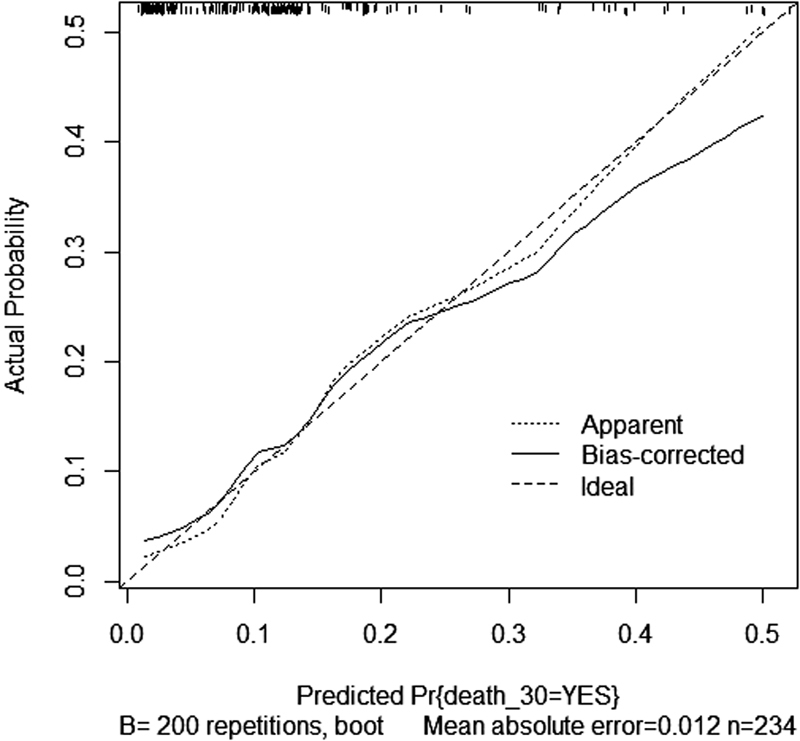
Calibration plot of the predictive model of death within 30 days since the diagnosis of acute PE. The plot was obtained after 200 bootstrap replicates to correct for optimism. The dashed diagonal line represents the ideal situation when predictive and observed probabilities of the outcome exactly coincide (slope = 1.00).

To estimate the predictive capability of the model, a ROC analysis was conducted. The AUC of the predictive model was 0.77 (95% CI: 0.67–0.87), indicating a good discriminative power. The AUC further increased to 0.85 (95% CI: 0.73–0.96) when D-dimer, categorized at the 75th percentile of its distribution in the whole cohort (i.e., 5,000 ng/mL), was added to a second predictive model together with ESC risk classes, age, and sex. The ROC curves of the two predictive models are shown in Fig. 2 . After bootstrap analysis (200 replicates), the AUC became 0.74 for the model without D-dimer and 0.80 for the model with D-dimer. The calibration plot sorted from the model containing also D-dimer levels, which compares the absolute probabilities of observed deaths within 30 days with those predicted by the model, is shown in Fig. 3 . The actual curve is very close to the theoretical ideal situation (perfect coincidence between observed and predicted probabilities) especially for low probabilities, indicating a good predictive performance of the model. The bias-corrected slope of the curve was equal to 0.79 (ideal slope: 1.00).

The capability of D-dimer to better discriminate low- and intermediate-low-risk patients has also been evaluated by comparing mortality with D-dimer levels. In particular, out of the 81 patients in the low ESC risk class, 64 had D-dimer below 5,000 ng/mL and 17 had D-dimer ≥5,000 ng/mL. One patient in each subgroup died within 30 days, for a mortality of 1.6% in the low-risk class with D-dimer less than 5,000 ng/mL and 5.9% in the low-risk class with D-dimer ≥5,000 ng/mL. Considering the 74 patients in the intermediate-low ESC risk class, 45 had D-dimer below 5,000 ng/mL (2 deaths) and 29 had D-dimer ≥5,000 ng/mL (5 deaths), for a mortality of 6.7% in the intermediate-low-risk class with D-dimer less than 5,000 ng/mL and 13.8% in the intermediate-low-risk class with D-dimer ≥5,000 ng/mL.

## Discussion

The prognostic model for early death after acute PE diagnosis proposed by the ESC in 2014 was validated in our cohort of patients. The 30-day risk of death increased 3.23 times in the intermediate-low-risk category, 5.55 times in the intermediate-high-risk category, and 23.78 times in the high-risk category, as compared with the low-risk category. According to the ROC analysis, the predictive model containing the four ESC risk classes, age, and sex showed a good discriminatory power, which further increased when D-dimer was added to the model. In this study, we observed an overall 10% death rate within 30 days since PE diagnosis, which is in line with other recent studies that showed a 30-day mortality rate ranging from 9 to 12%. 17 18 19 20 Concerning mortality divided by the subgroups, we observed a high mortality in the high-risk class (40%) and a low mortality in the low-risk class (2.5%), whereas the difference (5.3%) in mortality between intermediate-high-risk class and intermediate-low-risk class remains quite uncertain due to a wide confidence interval, in accordance with previous results. 8

We did not observe a relevant difference neither in male/female ratio nor in age between survivors and nonsurvivors. One would expect a higher mortality in the elderly, as considered in the PESI score and observed by Geibel et al. 21 In our cohort, the similar age distribution in survivors and nonsurvivors might be due to the narrow age range of our patients. In our patients, the prevalence of heart failure, cancer, and immobilization was higher in nonsurvivors than in survivors, according to previous findings by Huang et al. 17

The prevalence of dyspnea, chest pain, and syncope was similar in nonsurvivors as compared with survivors, while shock, tachypnea, hemoptysis, and DVT signs were more frequent in nonsurvivors. Our data are in agreement with the findings of Agrawal et al. 22 Nonsurvivors had lower values of systolic blood pressure and higher values of heart rate and respiratory rate than survivors. Lower values of blood pressure are frequently observed in PE patients with early mortality. 17 22 According to a meta-analysis of eight studies on 2,354 patients, sinus tachycardia was found to be predictive of 30-day mortality for PE. 23 Tachycardia and tachypnea are frequently described in PE patients not surviving at 30 days. 24 25 The evaluation of respiratory exchanges showed hypoxemia and low oxygen saturation in nonsurvivors, confirming a major impairment in this group of patients as reported also in previous studies. 22 25

Concerning the estimate of the predictive capability of the 2014 ESC model, our data confirm the efficacy of the model in stratifying the risk of mortality at 30 days, as recently assessed by Becattini et al. 8 Moreover, our data indicate that it is possible to increase the predictive ability of the model by adding D-dimer measured at PE diagnosis, which is an easy and cheap biomarker frequently evaluated in PE patients. The role of D-dimer as a diagnostic tool in patients with suspected PE and as a prognostic tool for VTE recurrence after anticoagulant therapy withdrawal is well recognized, 12 13 and previous studies have demonstrated its predictive role for mortality after PE diagnosis. 14 15 16 To the best of our knowledge, the addition of D-dimer to the predictive ESC model has been evaluated for the first time in our study. By adding D-dimer to a predictive model containing the four ESC risk classes, age, and sex gives the possibility to reclassify some individuals as low risk for early death, thus increasing the number of patients who can safely be discharged from the hospital for home treatment.

Some limitations of our study should be considered. First, the retrospective nature of the study limited the possibility to retrieve in all PE patients the information on some parameters for the correct definition of the ESC risk class for early mortality. However, most of the patients (86%) could be correctly classified and included in the prediction model, and the 38 patients who could not be included (all survivors) would be classified as intermediate-low- or low-risk patients, thus minimizing the possibility of having obtained biased results. Second, this is a single-center study, and the results might not be necessarily applicable to PE patients admitted to the emergency department of other hospitals. Nonetheless, all patients in this study were consecutively admitted to the emergency department of a large municipality hospital and covered a wide inclusion timeframe in which very few has changed in the approach to the patients, and the 10% rate of early death that we observed did not differ from that of other studies, thus reducing the possibility of a selection bias. Finally, all participants were white Caucasians, potentially limiting the generalizability of the study findings to other populations.

In conclusion, this study represents a good validation of the predictive model for early mortality after acute PE diagnosis proposed by the 2014 ESC guidelines. Adding D-dimer levels measured at PE diagnosis could further improve the performance of the prediction model, in particular, discriminating the mortality in low-risk classes. This could allow to identify patients who need hospitalization from those who can be safely discharged. An external validation to other cohorts of patients with acute PE is warranted to confirm our findings.
